# Research and Fabrication of High-Frequency Broadband and Omnidirectional Transmitting Transducer

**DOI:** 10.3390/s18072347

**Published:** 2018-07-19

**Authors:** Shaohua Hao, Hongwei Wang, Chao Zhong, Likun Wang, Hao Zhang

**Affiliations:** 1 School of Science, Beijing Information Science and Technology University, Beijing 100192, China; 15652868686@163.com (S.H.); 15652350954@163.com (H.Z.); 2 Sensing Technology Research Center, Beijing Information Science and Technology University, Beijing 100101, China; zclovelxm@163.com (C.Z.); wlikun@bistu.edu.cn (L.W.)

**Keywords:** composite materials, multimode coupling, curved-surface forming, high frequency, broadband, horizontal omnidirectional emission, underwater acoustic transducer

## Abstract

A wide-band cylindrical transducer was developed by using the wide band of the composite material and the matched matching layer for multimode coupling. Firstly, the structure size of the transducer’s sensitive component was designed by using ANSYS simulation software. Secondly, the piezoelectric composite ring-shaped sensitive component was fabricated by the piezoelectric composite curved-surface forming process, and the matching layer was coated on the periphery of the ring-shaped piezoelectric composite material. Finally, it was encapsulated and the electrodes were drawn out to make a high-frequency broadband horizontal omnidirectional water acoustic transducer prototype. After testing, the working frequency range of the transducer was 230–380 kHz, and the maximum transmission voltage response was 168 dB in the water.

## 1. Introduction

Sonar is an electronic system that detects and identifies underwater objects by means of acoustic waves. The underwater acoustic transducer is the key component of the sonar system. In recent years, the application of Unmanned Underwater Vehicle (UUV) has made rapid development of medium- and high-frequency underwater acoustic transducers. Generally, the high Q value of the high-frequency transducer results in its narrow working bandwidth and less information acquisition. However, due to the small beam angle of the planar transducer, the angle of the transmitting and receiving signals is limited. Thus, the hot topic of scholars at home and abroad is how to get wider bandwidths and larger beam angles of transducers.

In terms of expanding the bandwidth of the transducer, S. Cochran and others in Britain fabricated a transducer with a bandwidth of more than one octave by adding a matching layer to the 1-3 type piezoelectric composite material. Zhang Kai and others fabricated a dual matching layer high-frequency broadband transducer with a working range of 43–155 kHz. Although the above-mentioned transducer had expanded bandwidth, its beam angle was small, so it was difficult to realize the transmitting or receiving of large-angle underwater acoustic signals [1,2,3]. In terms of enlarging the transducer’s beam angle, MSI company had made 6 rows and 4 columns of arc-shaped transducer array; its working range was 8–16 kHz, and the horizontal opening angle was 150° degrees. Zhang Kai and others fabricated a high-frequency broadband transducer whose radial vibration frequency was 47.5 kHz and the working range was 40–80 kHz. Such transducers expanded the beam angle, but the bandwidth was relatively small [4,5,6].

Combined with most of the current research ideas, this paper combines the two most commonly used methods of expanding transducer bandwidth:

1. Composite materials

The concept of composite materials was proposed in the 1970s. It was defined as a material that combines a piezoelectric ceramic and a polymer in a certain communication mode, a certain volume or weight ratio, and a certain spatial geometric distribution. Adding a three-dimensionally connected polymer material to a one-dimensional piezoelectric material to fabricate a 1-3 type piezoelectric composite material can increase the loss of the transducing material and increase the bandwidth [7,8]. The mechanical quality factor of the transducing material can be expressed as:

(1) $$Q=\frac{\omega M}{R}\;$$

where Q is the mechanical quality factor of the transducing material, ω is the circular frequency, M is the equivalent mass of the transducing material, and R is the sum of the loss resistances of the transducing material. From Equation (1), it can be seen that increasing the loss of the transducing material can reduce the mechanical quality factor of the transducing material. In addition, the mechanical quality factor can be expressed as:

(2) $$Q=\frac{f_{r}}{\Delta f}$$

where $f_{r}$ is the resonant frequency of the transducing material and Δf is the frequency bandwidth of the 3 dB drop in conductance response. From Equation (2), it can be seen that the decrease of the mechanical quality factor of the transducing material expands the bandwidth of the transducing material. So, adding a flexible polymer to the piezoelectric material can extend the bandwidth of the transducing material.

2. Adding a matching layer

When no matching layer is added, the impedance of the water load is the impedance of the surface of the transducing material. After adding a matching layer with a specific acoustic characteristic impedance to the acoustic radiation surface of the transducing material, the impedance of the water load has the impedance generated by the matching layer in addition to the impedance of the surface of the transducing material. The difference in the two impedances produces two resonant frequencies. Adjusting the thickness of the matching layer brings the two resonant frequencies close enough to couple to expand the working bandwidth of the transducing material [9,10].

At the same time, using a curved-surface forming process to fabricate a circular piezoelectric composite can increase the beam angle of the transducer.

The PZT-5A (produced by Risheng Electronics Co., Ltd., Kunshan, China) was selected as a piezoelectric material to fabricate a transducer. At present, in addition to the traditional piezoelectric materials, the high-performance requirements of the transducers have led to the development of new piezoelectric materials, including piezoelectric composites, relaxed ferroelectric single crystals, etc. At this stage, the relaxation ferroelectric single crystal has been a hot spot due to its high piezoelectric coefficient ($d_{33}$) and high mechanical quality factor ($Q_{m}$) [11,12]. The transducer based on the relaxed ferroelectric single crystal can increase the sensitivity by 12 dB, the bandwidth by 2–3 times, and the sound source level by 12 dB. In terms of the conventional PZT piezoelectric ceramic, it is relatively hard and has the ability to exert and withstand a large stress in physical properties. From a chemical point of view, it is inert and unaffected by moisture and other atmospheric conditions. The manufacturing method is also relatively simple, and as a transducing material, it also has excellent piezoelectric properties. Compared with the relaxed ferroelectric single crystal, the PZT-5A piezoelectric material has a higher Curie temperature, so its temperature stability is high, the aging rate is small, the time constant is large, the manufacturing cost is low, it can be molded in a large area, and commercialized application is more mature. Compared to the transducers designed here, the cost of preparing a transducer using the same size single crystal material can be 5–6 times higher. A relaxation ferroelectric single crystal PMN-PT29 material (produced by Materials Research Institute, Pennsylvania State University, State College, PA, USA) was selected and compared with the piezoelectric ceramic PZT-5A material. The material parameters of the two are shown in Table 1:

For transmitting transducers, mechanical loss (larger $Q_{m}$) is generally required to improve the efficiency of the emission. However, sometimes it needs to increase the bandwidth and requires a smaller $Q_{m}$ material. In summary, PZT-5A was chosen as the transducer material to fabricate the transducer. Furthermore, the PZT-5A material was used to prepare the 1-3 type piezoelectric composite. Compared with pure PZT-5A piezoelectric ceramics, the hydrostatic pressure constant $g_{h}$ is 1–2 orders of magnitude higher. Due to the addition of the flexible polymer, the equivalent density of the transducing material is reduced, and the acoustic medium has a good acoustic matching. As a “soft” piezoelectric material, PZT piezoelectric ceramics can be prepared into a desired shape by adding a flexible polymer. This soft nature makes it more resistant to vibration and mechanical shock, which can increase the service life of the transducer in a complex seawater environment.

From the beginning to the present, piezoelectric composite materials have been a research hotspot. Many scholars did a large number of theoretical and experimental research on piezoelectric composites, and also tested the impact of ceramic volume fraction on the performance of piezoelectric composites. For transducer applications, the key material parameter is the electromechanical coupling factor and variation of charge constant, which are closely related to device bandwidth and sensitivity. For example, Smith W.A. and Auld B.A. et al. studied the impact of different piezoelectric ceramic volume fractions on electromechanical coupling factor. The results showed that the electromechanical coupling coefficient shows an upward trend within 20% of the volume fraction; it remains stable in the range of 20% to 80%; and it shows a downward trend in the range of 80% to 100% [13]. Chan H.L.W. and Unsworth J. et al. studied the impact of different piezoelectric ceramic volume fractions on the piezoelectric charge constant $d_{33}$. The results showed that the piezoelectric charge constant $d_{33}$ shows an upward trend within 40% of the ceramic volume fraction, and basically stabilizes after 40% [14]. T.R. Gururaja et al. tested the impact of different volume fractions on mechanical quality factor. It was found that the composite material has a lower mechanical quality factor than the pure piezoelectric ceramic material, which is advantageous for expanding the bandwidth of the transducer [15]. Based on the above studies, we found that the volume fraction of piezoelectric ceramics is in the range of 40–60%, and its comprehensive performance is optimal. Therefore, we chose to prepare a 1-3 type piezoelectric composite with a piezoelectric ceramic volume fraction of 50%.

Finally, the experimental results show that the piezoelectric composite transducer fabricated in this paper has achieved the target of high-frequency wideband and wide beam angle. The design theory and fabrication process will greatly promote the study of the extended bandwidth and the beam angle of the high-frequency transducer.

## 2. Structure of a High-Frequency Wideband Composite Cylindrical Transducer

**Figure 1:**
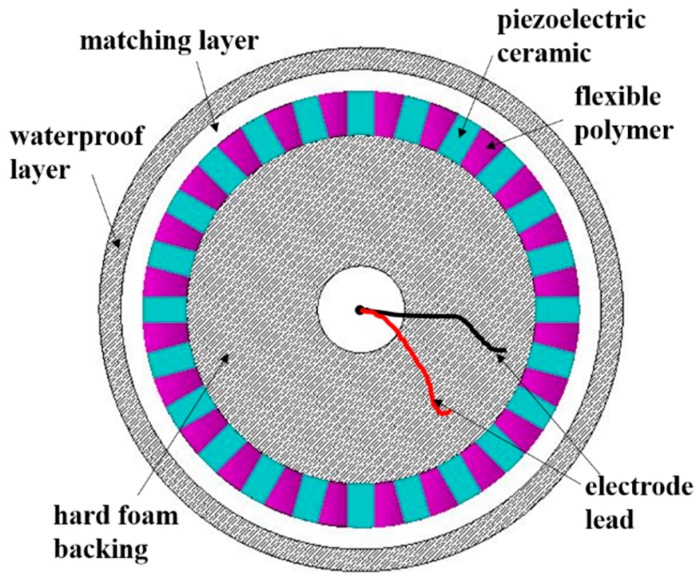
The structure of the high-frequency broadband composite cylindrical transducer.

The structure of the high-frequency broadband composite cylindrical transducer is shown in Figure 1.

**Figure 2:**
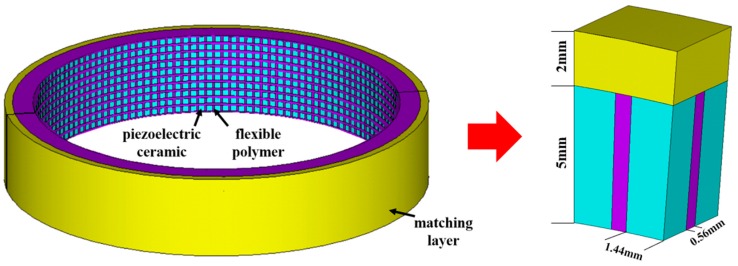
The structure of 1-3 type piezoelectric composite material with matching layer.

It consists of piezoelectric composite material, matching layer, hard foam backing, waterproof sound transmission layer, and electrode lead. The piezoelectric composite material is composed of piezoelectric ceramic and flexible polymer. One-dimensional connected piezoelectric ceramic is arranged in a three-dimensional connected flexible polymer to form a 1-3 type piezoelectric composite. Its advantage is that it has a purer thickness vibration mode and can realize the transducer working in the high-frequency range. At the same time, this composite structure provides the possibility for the curved-surface forming of the transducer. Therefore, we use the 1-3 type piezoelectric composite material as the sensitive component of the transducer to realize the performance of high-frequency broadband and wide beam angle. Adding a matching layer to the acoustic radiation surface of the sensitive component forms two kinds of vibration modes. Adjusting the thickness of the matching layer enables the coupling of two vibration modes in the water to expand the bandwidth of the transducer. The matching layer can also produce the effect of prestress, which makes the amplitude difference of each point on the vibration surface smaller. The most important part of the transducer is the 1-3 type piezoelectric composite material with matching layer. Its structure with the individual ceramic dimensions and the dimensions of the polymer part is shown in Figure 2.

**Figure 3:**
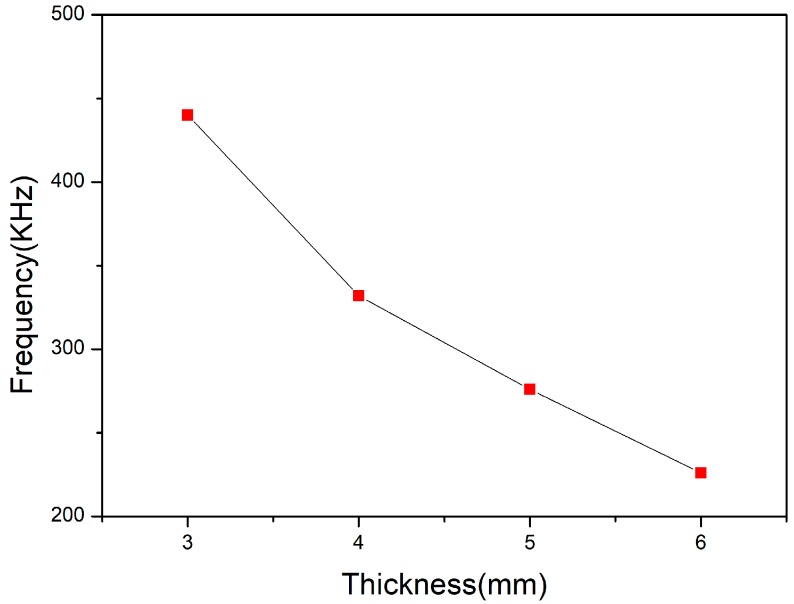
The variation curve of thickness resonance frequency with ceramic thickness.

Piezoelectric ceramics are used as active components and their size determines the parameters of the transducer. Since the thickness vibration mode of the piezoelectric ceramic is used in this design, the influence of the thickness of the piezoelectric ceramic on the frequency of the transducer is mainly considered. The finite element simulation of piezoelectric ceramics with different thicknesses was carried out by ANSYS software, and the variation curve of thickness resonance frequency with ceramic thickness was obtained as shown in Figure 3:

It can be seen from Figure 3 that as the thickness increases, the thickness resonance frequency decreases. A piezoelectric ceramic of 5 mm thickness was selected based on design requirements near 300 kHz.

**Figure 4:**
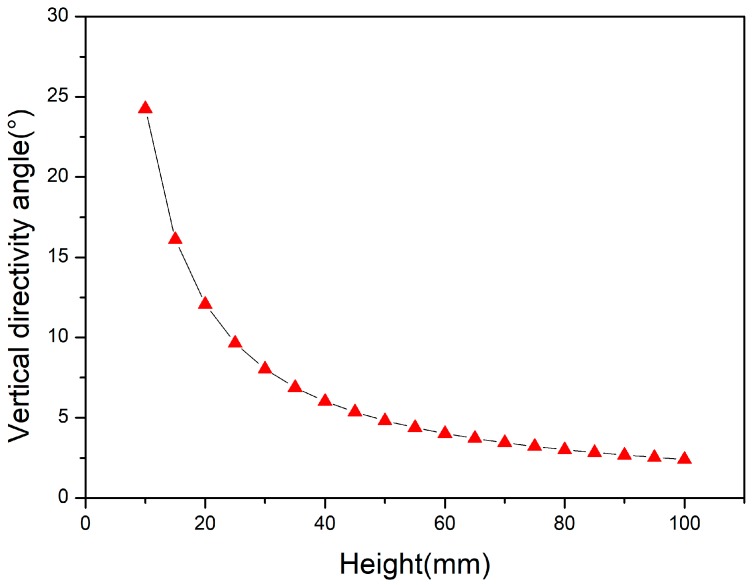
The variation of the directivity angle of the vertical direction with the vertical direction.

The height of the transducer is determined by the directivity requirements of the transducer in the vertical direction. For composite materials, the vertical direction directivity calculation formula is shown in Equation (3):

(3) $$DI=\frac{\sin\left(\pi h/\lambda \sin\theta \right)}{\pi h/\lambda \sin\theta }$$

where DI is the directivity angle in the vertical direction, h is the height in the vertical direction, *λ* is the wavelength of the sound wave in the water, and *θ* is the calculation range. The variation of the directivity angle of the vertical direction with the vertical direction can be obtained by Matlab software calculation, as shown in Figure 4:

The vertical directivity angle required for the transducer of this design is about 5°, so the height selected by Figure 4 is 50 mm.

## 3. Finite Element Analysis

### 3.1. Finite Element Simulation of Sensitive Component in the Air

The harmonic response of the circular sensing component structure in the air was analyzed by ANSYS finite element simulation software. Firstly, the material parameters of the piezoelectric and polymer material and matching layer were set up. The parameters of PZT-5A were used for piezoelectric materials and the parameters of 618 epoxy resin were used for polymer and matching layer. The reason for selecting PZT-5A piezoelectric ceramics is as described above. Epoxy resin is one of the most widely used thermosetting resins in polymer materials. The most commonly used one is bisphenol A epoxy resin, which is characterized by: thermoplastic resin, good processability, high strength and bonding strength of the cured product, good corrosion resistance and electrical insulation, and small volume shrinkage after curing [16,17]. For the preparation of curved composite materials, liquid bisphenol A epoxy resin is required. The common domestic liquid bisphenol A epoxy resin and the main parameters are shown in the following Table 2:

The higher the epoxy value, the lower the viscosity and the greater the brittleness after curing. The flexible polymer required for the composite material designed in this paper must ensure a certain degree of brittleness and a certain degree of toughness. By comparison, the 618-type epoxy resin has a moderate epoxy value and a moderate viscosity. Therefore, the 618-type epoxy resin was selected.

**Figure 5:**
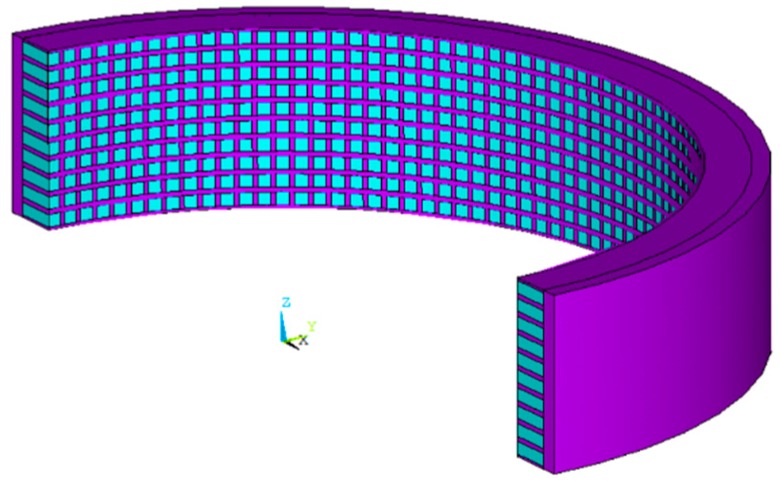
The structure of the simulation model.

The parameters of PZT-5A are density ρ, dielectric constant matrix $\varepsilon^{S}$, piezoelectric constant matrix e, and elastic constant matrix $c^{E}$. The specific values are shown in Table 3:

The parameters of the epoxy resin are density ρ, Young’s modulus E, and Poisson’s ratio δ. The specific values are shown in Table 4:

Secondly, a model of circular ring sensing component was established. To save computing time, the model was designed to be 180° arc with a thickness of 5 mm. The model diagram is shown in Figure 5. The ceramic volume of the 1-3 type composite without adding matching layer accounted for 50% of the total volume. Then, the model was meshed into finite elements. Finally, the 1 V voltage was loaded on the outer surface of the arc and 0 V voltage was loaded on the inner surface of the arc. Symmetric boundary conditions were loaded in a height direction. Harmonic response analysis was carried out in the air from 200 kHz to 400 kHz frequency range [18]. The damping coefficient was set to 0.02.

**Figure 6:**
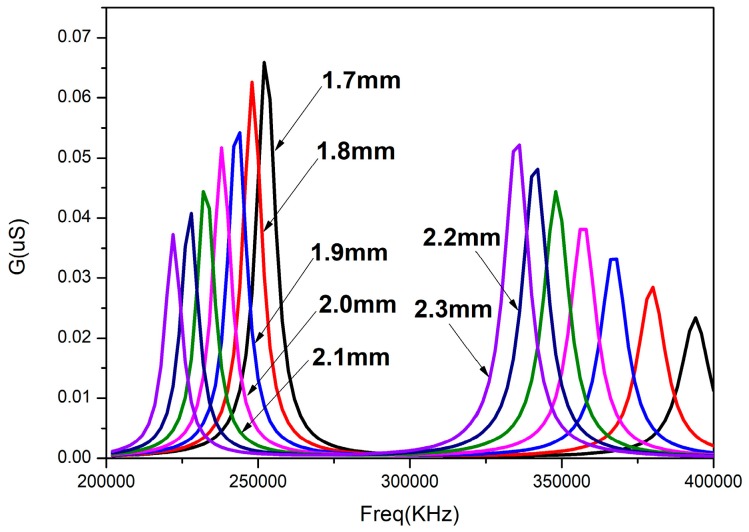
The conductance curves of sensitive component with different thickness matching layers.

**Figure 7:**
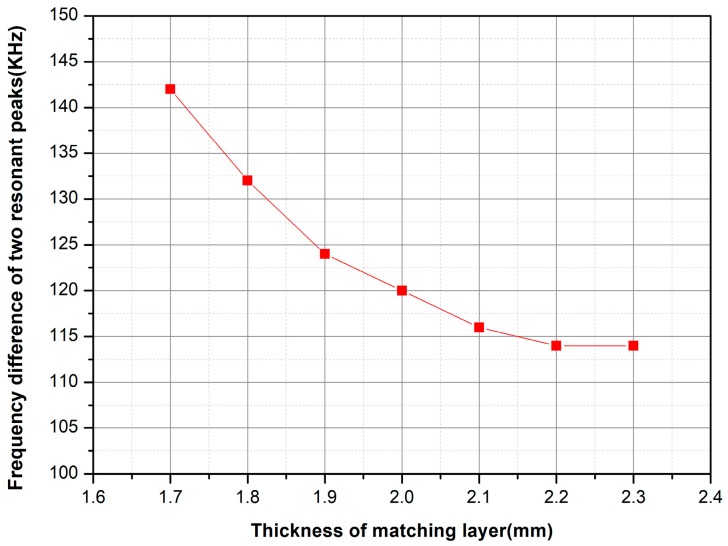
The difference of the resonant peak value of the two vibration modes with different matching layer thicknesses.

The design of the matching layer was based on the quarter-wavelength theory [19,20], and its thickness can be calculated by the following formula:

(4) $$t=\frac{1}{4}\lambda$$

where *t* is the thickness of the matching layer and λ is the wavelength of the sound wave in the matching layer. The matching layer was made of epoxy resin, and the wavelength of the acoustic wave in the epoxy resin was about 8 mm, so the thickness of the epoxy matching layer was selected to be about 2 mm. The arc model with different thickness of epoxy resin matching layer was established and simulated by ANSYS finite element simulation software. The conductance curve with frequency of the sensitive component with different matching layer thicknesses from 1.7 mm to 2.3 mm is shown in Figure 6. The difference of the resonant peak value of two vibration modes with different matching layer thicknesses is shown in Figure 7.

It can be seen that two vibration modes are formed after adding the matching layer in Figure 4. The resonant peaks of the matching layers with different thicknesses are also different. The frequency difference between the two resonant peaks increases as the thickness of the matching layer increases. The conductance of the first resonance peak decreases, and the conductance of the second resonant peaks increases. From Figure 6, it can be concluded that the difference of the two resonant peaks decreases with the increase of the thickness of the matching layer. The damping coefficient in the air is small and cannot be coupled to a resonant peak. However, the damping coefficient in the water is larger than that in the air and the two vibration modes can be coupled to expand the bandwidth of the transducer. At the same time, the matching layer whose thickness is 2 mm is easier to be fabricated. So, the thickness of the matching layer to be added is 2 mm.

**Figure 8:**
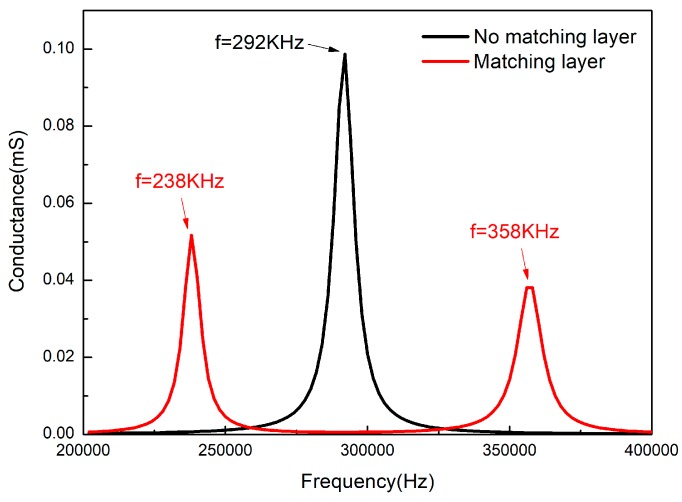
The conductance curves obtained from the simulation of two models.

Based on the above ideas, a composite material model without matching layer and a composite material model with 2 mm thick epoxy resin matching layer on the outside surface were established. The harmonic response of the two models were calculated and the conductance curves are shown in Figure 8.

### 3.2. Finite Element Simulation of Sensitive Component in the Water

**Figure 9:**
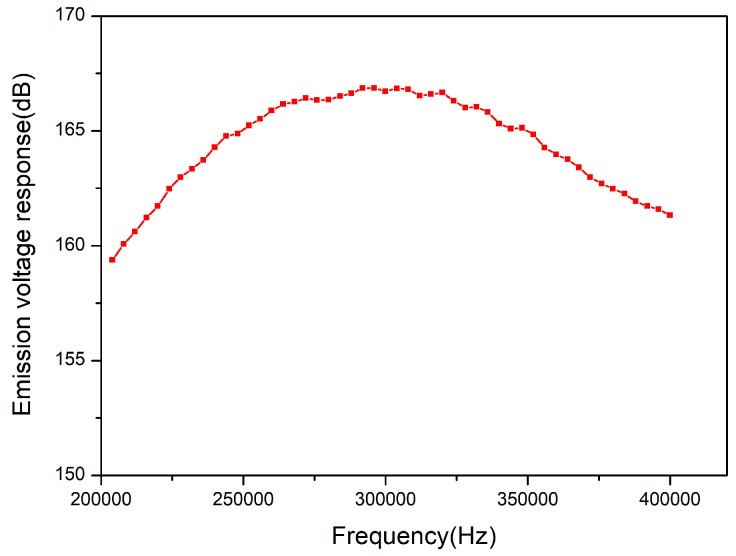
The transmitting voltage response curve obtained from the model simulation.

Based on the simulation steps of sensitive component in the air, the near-field water, far-field water, and boundary water models were added to simulate the sensitive component in the water environment. The parameters set by the simulation of the water environment include density, sound velocity, and boundary admittance as shown in Table 5:

In order to save computing time, we continued to simplify the simulation model. The model was 1/144 of the actual transducer. The height direction and the circumferential direction were respectively loaded with symmetrical boundaries and the damping coefficient was increased to 0.04. Finally, harmonic response analysis was carried out, and the transmitting voltage response curve calculated is shown in Figure 9.

It is known that the maximum value of transmitting voltage response which was simulated in the water is 166.8 dB in Figure 9. The −3 dB bandwidth of the transmitting voltage response is 128 kHz. It shows that the transducer bandwidth can be expanded by adding 2 mm thick matching layer on the composite surface.

## 4. Fabrication and Testing of High-Frequency Broadband Circular Sensitive Element

**Figure 10:**
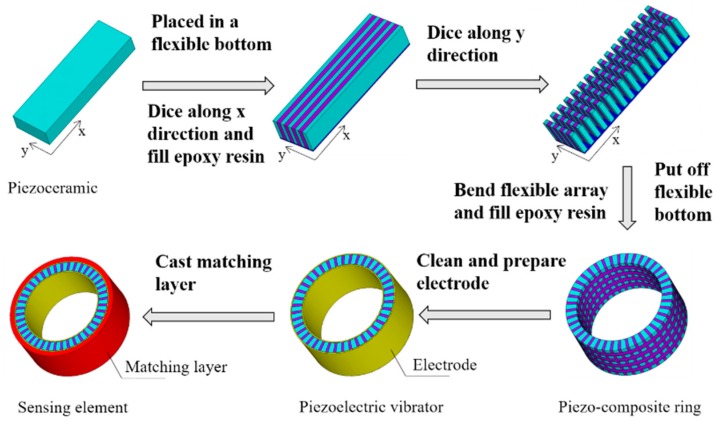
The fabrication flowchart of circular-shaped sensitive element.

PZT-5A piezoelectric ceramic blocks which is produced from the Institute of Acoustics of the Chinese Academy of Sciences were used as piezoelectric materials. The thickness vibration mode of piezoelectric ceramic was adopted, so the thickness direction polarization was chosen. The ring-shaped sensitive component was fabricated by an improved cut-filling method. Its fabrication flowchart is shown in Figure 10.

**Figure 11:**
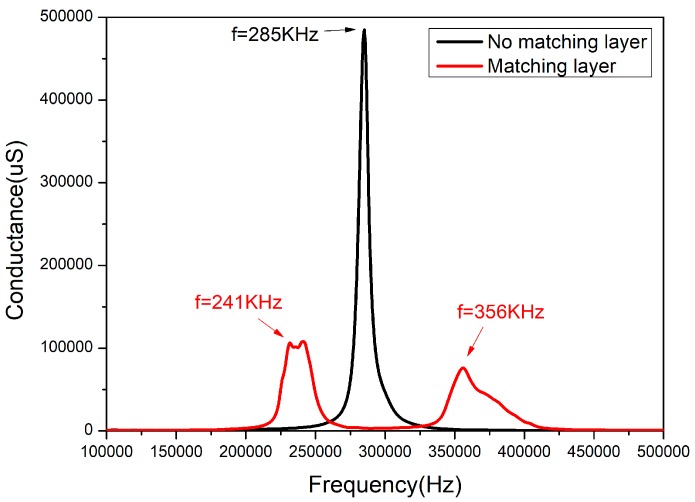
The conductance curve obtained by the actual testing of two models.

1-3 type piezoelectric composite ring was fabricated according to the above fabrication process, and the electrode was coated inside and on the outside surface. The conductance was tested by the Agilent-4294A impedance analyzer (produced by Agilent Technologies, Inc., Santa Clara, CA, USA) in the air. The epoxy resin matching layer with a thickness of 2 mm was added to the outer surface of the composite. After curing, the conductance was also tested by the Agilent-4294A impedance analyzer in the air. The conductance curve of the two models are shown in Figure 11.

It can be seen from Figure 11 that the resonance peak of the composite material without matching layer appears at 285 kHz and its bandwidth is only 8.5 kHz. However, after adding a 2 mm thick epoxy matching layer, there are two resonant peaks in the conductance curve, the frequencies of which are 241 kHz and 356 kHz, respectively. Matching layer and composite material produce two vibration modes in the air and two vibration modes are coupled in the water. The deviation between experiment results and simulation results is less than 3%, which meets the design requirements.

## 5. Fabrication and Testing of Transducer

### 5.1. Fabrication of High-Frequency Broadband Circular Transducer

**Figure 12:**
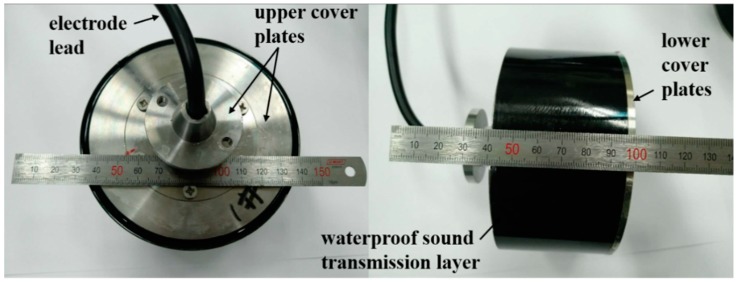
The high-frequency broadband circular transducer.

The high-frequency broadband circular transducer is mainly composed of sensitive component, rigid foam lining, upper and lower cover plates, waterproof sound transmission layer, and electrode lead. The rigid foam lining was embedded in the inside surface of the sensitive component and bonded with epoxy resin. The electrode lead was passed through the upper cover plate and drawn from the circular hole in the middle of the upper cover plate. Then, the sensitive component was put into the mold used to pour the waterproof sound transmission layer. The waterproof sound transmission layer used polyurethane with similar properties to the water. The polyurethane was slowly poured into the mold and solidified for 12 h in the 60 °C temperature chamber. Finally, the gap between the circular hole and the electrode lead was sealed. The fabricated cylindrical transducer is shown in Figure 12.

During the fabrication of sensitive component and transducer, it was necessary to pay attention to the cleaning of the mold to ensure that there was no impurity in the filling material to affect the performance of the transducer. The process of curved-surface forming was rather complicated, so in the process of perfusion, we had to grasp the operation time in every step to ensure the consistency of ceramic arrays. The outer dimension of the circular transducer was Ф 112 mm × 60 mm in this experiment, in which the composite material element height of the transducer was 50 mm.

### 5.2. Testing of High-Frequency Broadband Circulars Transducer

**Figure 13:**
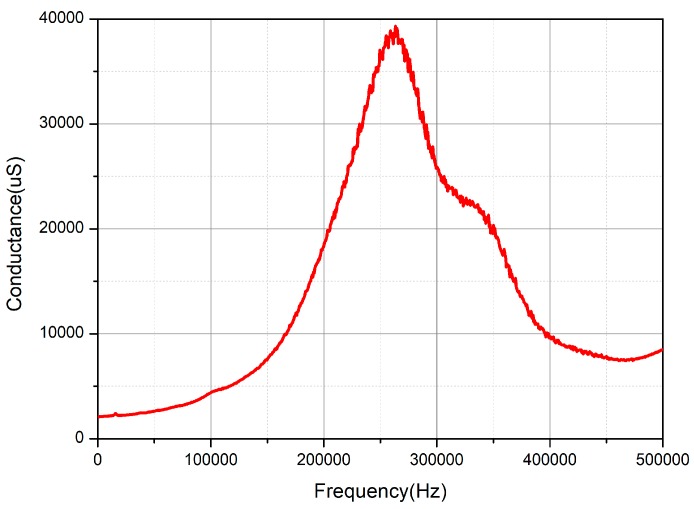
The conductance curve of transducer in the water.

The impedance performance of the transducer in the water was measured by the Agilent 4294A impedance analyzer after the fabrication of the transducer. The conductance curve in the water is shown in Figure 13.

As can be seen from Figure 13, there is only one resonant peak in the conductivity curve, which is due to the larger damping coefficient in the water than in the air, and the two resonant peaks in the water are coupled to a wide band of resonant peaks. Its working frequency range is from 203 kHz to 351 kHz.

**Figure 14:**
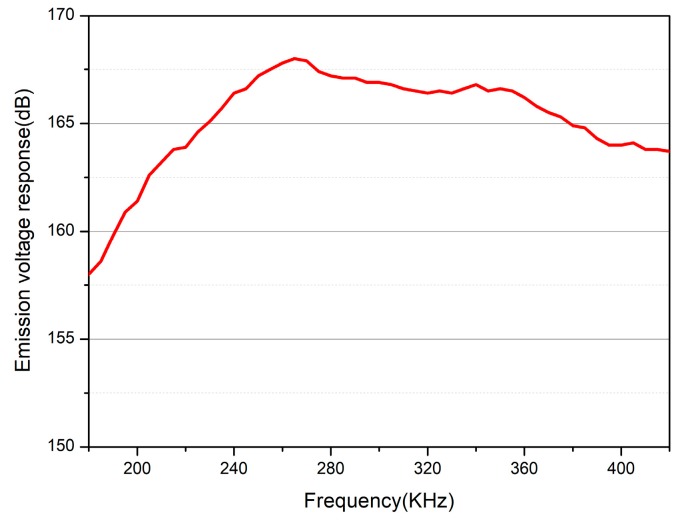
The emission voltage response curve of transducer in the water.

The bandwidth of the transducer can focus on not only the bandwidth of its conductance curve, but also its bandwidth in the transmitting voltage response curve [21,22]. The transmitting voltage response and directivity of the transducer in the water were measured by the electroacoustic measurement system of the underwater transducer. The instruments included in the electroacoustic measurement system are shown in Table 6:

The range of measurement is set up from 180 kHz to 420 kHz. The transmitting voltage response curve with frequency is shown in Figure 14. The maximum transmitting voltage response is 168 dB, and the working frequency range is 230–380 kHz.

Compared with the actual test and simulation results of the emission voltage response, the maximum emission voltage response is basically the same, and the bandwidth is different. Since the bandwidth is related to the damping coefficient, the damping coefficient in the simulation is set to a fixed value, and in fact, the damping coefficient changes with the frequency, so there is an error. However, the actually prepared transducer has a transmission voltage response bandwidth that has reached half of the octave.

**Figure 15:**
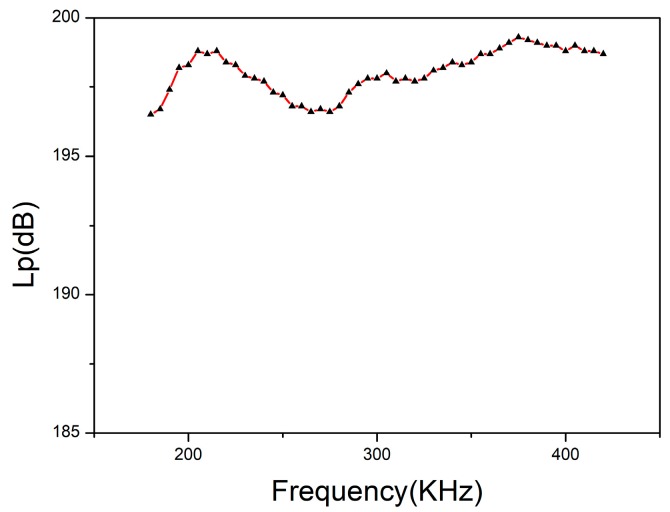
The variation of the emission sound source level with the frequency.

The sound source level $L_{p}$ is generally used to describe the strength of the acoustic signal emitted by the active sonar, that is, the sound power level of the single-frequency transmission. The emission sound source level curve can be obtained by the electroacoustic measurement system as shown in Figure 15:

It can be seen from Figure 3 that when the highest voltage applied across the transducer is 30 V, the maximum sound source level can reach 199 dB.

**Figure 16:**
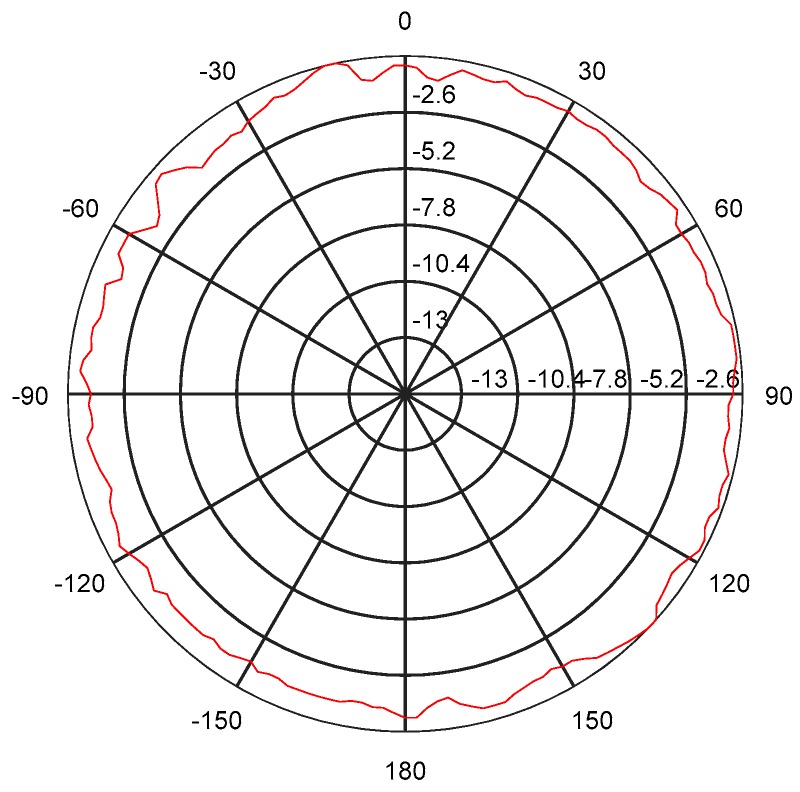
The directivity curve of transducer in the water.

The measurement result of the transducer’s directivity is shown in Figure 16. It is known that the transducer has omnidirectional directivity in one-dimensional direction in Figure 16, and the fluctuation is more stable within −3 dB.

After testing, the transducer parameters were obtained, and some advanced curved surface composite transducers were compared. For example, the USA MSI provides communication sonar for the USA Navy, and M.S. Martins et al. produced a high-frequency wide-beam PVDF transmitting transducer for underwater wireless communication and other fields [23,24]. The specific parameters are compared as in Table 7:

Through the above comparison, it is found that the transducer designed in this paper has lower operating frequency, bandwidth of 1/2 octave, higher response voltage, and omnidirectional emission of sound waves in the horizontal direction compared with other advanced transducers. The advantages of this transducer are obvious.

## 6. Discussion and Conclusions

In this paper, the bandwidth of transducers was expanded by means of composite material and multimode coupling, and a curved-surface forming process was adopted. Finally, an underwater acoustic transducer was fabricated with a working frequency band of 230–380 kHz. The maximum transmission voltage response in the frequency band was 168 dB. It can be launched omnidirectionally in one-dimensional direction. The test results are basically consistent with the simulation results. Compared with the same type of transducer, the working band width of the transducer is about doubled. The transducer can be applied to underwater vehicles, deep sea target detection, fine imaging, and so on.

## Figures and Tables

**Table 1:** Comparison of the parameters of PZT-5A and PMN-PT29.

Material	$T_{c}\;\left({}^{\circ}C\right)$	$E_{c}\;\left(kV/cm\right)$	$\varepsilon_{r}$	tanσ	$d_{33}\;\left(pm/V\right)$	$Q_{m}$
**PZT-5A**	360	15	1700	0.02	380	75
**PMN-PT29**	135	2.3	5400	0.004	1540	150

**Table 2:** The common domestic liquid bisphenol A epoxy resin and the main parameters.

Epoxy Resin Model	Epoxy Value (mol/100 g)	$\mathrm{Epoxy}\;\mathrm{Equivalent}\;\left(g\cdot mol^{-1}\right)$
616	0.52–0.56	179–192
618	0.48–0.54	185–208
6101	0.41–0.47	213–244
637	0.30–0.40	250–330

**Table 3:** The specific values of PZT-5A.

$\rho \;\left(kg/m^{3}\right)$	$\varepsilon^{S}$	$e\;\left(c/m^{2}\right)$	$c^{E}\;\left(10^{10}N/m^{2}\right)$
7750	$\varepsilon_{11}=830$	$e_{11}=15.8$	$c_{11}=11.1$ $c_{33}=12.1$
	$\varepsilon_{22}=916$	$e_{31}=-5.4$	$c_{12}=7.52$ $c_{44}=2.11$
	$\varepsilon_{33}=916$	$e_{24}=12.3$	$c_{13}=7.52$ $c_{55}=2.26$

**Table 4:** The specific values of epoxy.

$\rho \;\left(kg/m^{3}\right)$	E (Pa)	δ
$1.25\times 10^{3}$	$3.6\times 10^{9}$	0.35

**Table 5:** The specific values of water environment.

Material	$\rho \;\left(kg/m^{3}\right)$	Sonic Velocity (m/s)	Boundary Admittance (S)
**Near-field water**	1000	1480	0
**Far-field water**	1000	1480	0
**Boundary water**	1000	1480	1

**Table 6:** The instruments included in the electroacoustic measurement system.

Instrument	Brand	Model
15 MHz Multifunction Synthesizer	NF Corporation (Kanagawa Japan)	WF1946B
Voltage and Current Sensor	USA Instruments Inc. (San Diego, CA, USA)	VIT-13
Kilowatt Amplifier	USA Instruments Inc. (San Diego, CA, USA)	M4/M8
Dual Channel Programmable Filter	NF Corporation (Kanagawa Japan)	3628
Mixed Signal Oscilloscope	Agilent Technologies, Inc. (Santa Clara, CA, USA)	MSO6032A

**Table 7:** Comparison of the parameters of the transducer designed in this paper with other advanced transducers.

Transducer	Resonant Frequency (kHz)	Bandwidth (kHz)	Transmit Voltage Response (dB)	Horizontal Beam Angle (°)
Communication sonar (MSI)	900	8–16	137	150
PVDF transmitting transducer	750	250	150	>75
Broadband and omnidirectional transmitting transducer	300	150	168	360
